# Immunogenicity and Protective Response Induced by Recombinant Plasmids Based on the BAB1_0267 and BAB1_0270 Open Reading Frames of *Brucella abortus* 2308 in BALB/c Mice

**DOI:** 10.3389/fcimb.2016.00117

**Published:** 2016-09-29

**Authors:** Leonardo A. Gómez, Francisco I. Alvarez, Pablo A. Fernández, Manuel R. Flores, Raúl E. Molina, Roberto F. Coloma, Angel A. Oñate

**Affiliations:** Laboratory of Molecular Immunology, Department of Immunology, Faculty of Biological Sciences, Universidad de ConcepciónConcepción, Chile

**Keywords:** brucellosis, DNA vaccines, zinc-dependent metalloproteinase, Src homology 3 domain, interferon gamma

## Abstract

Immunogenicity induced by recombinant plasmids based on the BAB1_0267 and BAB1_0270 open reading frames (ORFs) of *Brucella abortus* 2308 was evaluated. Bioinformatics analyses indicate that the BAB1_0267 and BAB1_0270 ORFs encode a protein with a SH3 domain and a Zn-dependent metalloproteinase, respectively. Both ORFs have important effects on intracellular survival and replication of *B. abortus* 2308, mediated via professional and non-professional phagocytic cells. Our results show that immunization with the recombinant plasmid based on the BAB1_0267 ORF significantly increases the production of IgG1, levels of IFN-γ and the lymphoproliferative response of splenocytes. However, BAB1_0267 did not provide significant levels of protection. The plasmid based on the BAB1_0270 significantly increased IgG2a production, levels of IFN-γ and TNF-α, and the lymphoproliferative response of splenocytes. These results demonstrate that immunization with the BAB1_0270 derived recombinant plasmid induce a Th1-type immune response, correlated with a heightened resistance to *B. abortus* 2308 infection in mice. It is concluded that the Th1-type immune response against bacterial Zn-dependent metalloproteinase induces a protective response in mice, and that pV270 recombinant plasmid is an effective candidate microbicide against brucellosis.

## Introduction

Brucellosis, caused by *Brucella* spp., a Gram-negative coccobacillus, is a zoonosis of worldwide occurrence. This disease is associated with high morbidity in animals and humans, leading to important economic losses and public health problems in many countries (Seleem et al., 2010). Each *Brucella* species has a high degree of host specificity, but most can infect humans; *B. melitensis, B. abortus*, and *B. suis* being the most pathogenic (He, 2012). *B. abortus* infects mainly bovines of reproductive age, causing abortion, and infertility. In humans, the infection is characterized by undulant fever during its acute phase and localization of the pathogen in several organs during its chronic phase and, if not treated, it can become an invalidating or even fatal disease (Franco et al., 2007; Seleem et al., 2010).

The chronicity of brucellosis is related to the intracellular facultative lifestyle of *Brucella* organisms, which are able to live in different cell types, including macrophages and dendritic cells. It has been demonstrated that *B. abortus*, after entering a cell, avoids the last step of the phagolysosome pathway (i.e., fusion with a lysosome) by residing within a unique organelle, the *Brucella*-containing vacuole, that is derived from the endoplasmic reticulum. After maturation, assisted by the VirB Type IV secretion system of *Brucella*, the vacuole becomes permissive of replication (Comerci et al., 2001). In addition, the mature vacuole strategically modulates specific aspects of the immune response, making the host tolerant and therefore achieving the chronicity of the disease (Martirosyan et al., 2011). *Brucella* spp. also do not produce classical virulence factors, such as capsules, exotoxins, secreted proteases, fimbriae, phage encoded toxins, or virulence plasmids, which contributes to their adaptive success (Lamontagne et al., 2010).

Open reading frames (ORFs), present within genomic islands (GIs), are known to encode several virulence factors in brucellosis. In particular, some GI3 ORFs are important to *B. abortus* 2308 virulence (Céspedes et al., 2012; Salcedo et al., 2013). Induced mutations in the GI3 BAB1_0267 and BAB1_0270 ORFs of *B. abortus* 2308 have indicated their role in intracellular survival and replication of this pathogen in professional and non-professional phagocytes (Ortiz-Román et al., 2014). Furthermore, bioinformatics information indicates that BAB1_0267 codifies a protein with a Src homology 3 (SH3) domain. These domains are present in a wide variety of intracellular and membrane proteins (Bakal and Davies, 2000). It has been postulated that SH3 domains promote bacterial survival intracellularly by modulating SH3 domain associated signaling pathways of the eukaryotic cell and/or promoting invasion by binding to receptors present on the host cell (Whisstock and Lesk, 1999; Bakal and Davies, 2000). This latter function has been described by Bougneres (Bougneres et al., 2004), who demonstrated that *Shigella* invades eukaryotic cells by means of SH3 domains. BAB1_0270 ORF encodes a Zn-dependent metalloproteinase, a member of the widely distributed Zn-metallopeptidase family in bacteria. This protein serves as virulence factor in many pathogens (Barrett et al., 2004; Bonis et al., 2010; Cafardi et al., 2013; Menon and Govindarajan, 2013). Some bacteria like *Mycobacterium tuberculosis* express Zn-metallopeptidases for immune evasion purposes, while *Helicobacter pylori* use them for colonization and acquire morphological changes (Master et al., 2008; Bonis et al., 2010). Given that proteins with SH3 domains and Zn-metalloproteases are important for bacterial virulence, we hypothesize that *B. abortus* 2308 BAB1_267 and BAB1_0270 ORFs are potential candidates for developing new vaccines against Brucellosis.

To prevent bovine brucellosis, immunization with the vaccinal *B. abortus* RB51 and *B. abortus* S19 strains has been implemented. Although, both vaccines have been effective they cannot eradicate the pathogen and they are pathogenic for humans (Schurig et al., 2002). It is therefore necessary to develop an effective and safe vaccine able to trigger host immunity against *B. abortus*. A DNA vaccine that safely expresses immunogens protecting against *Brucella* infection could be more effective for controlling brucellosis. Immunization with DNA vaccines activates cell-mediated immunity (Shedlock and Weiner, 2000). The infectious agents are eliminated by high levels of IFN-γ and TNF-α, produced by activated Th1 CD4^+^ lymphocytes (Golding et al., 2001). Among the cytokines secreted, IFN-γ is pivotal in the clearance of intracellular pathogens and thus is required to eliminate *B. abortus* (Murphy et al., 2001). The aim of this study is to use *B. abortus* 2308 BAB1_0267 and BAB1_0270 ORFs to construct DNA vaccines and to evaluate their effect on the immune response in a BALB/c mouse model.

## Materials and methods

### Animals

Ten-week old female isogenic BALB/c mice were obtained from the Instituto de Salud Pública (Santiago, Chile). The animals were kept at the Laboratory of Molecular Immunology (Department of Microbiology, Faculty of Biological Sciences, Universidad de Concepción, Chile) and after arrival were randomly distributed into experimental and control groups and acclimated. The mice were kept under controlled temperature and fed with commercial pellets and water *ad libitum*. All regulations from the Institutional Bioethics Committee of the Faculty of Biological Sciences, Universidad de Concepción, were fulfilled. The Bioethics and Security committee of the Faculty of Biological Sciences in the Universidad de Concepción approved this study. All efforts were made to minimize animal suffering.

### Construction of recombinant plasmids

Vaccines were constructed by cloning the *B. abortus* BAB1_0267 and BAB1_0270 ORFs into the pVAX1 vector (Invitrogen). To achieve this, the ORFs were amplified by means of the polymerase chain reaction (PCR) using primers design in accordance with the appropriate sequences available at GenBank (CAJ10223.1 and CAJ10226). The final primer sequences, including the cutting sites for *Bam*HI and *Xho*I, were: BAB1_0267 forward: 5′wordGCCGGATCCGCCACCATGTTGTCGAAGGCGAAAAACTGG3′ and reverse: 5′wordTTTCTCGAGCTAAACCACCCAGAGCGGT3′. The final sequence to BAB1_0270 was forward: 5′word-GCCGGATCCGCCACCATGAGCAGTCAGAATTACGTTGTC-3′, and reverse: 5′word-GCCCTCGAGTCAGATCCCTTTTTTATTGATCCA-3′. PCR product was cloned into the pVAX1 vector using T4 DNA ligase (Promega, Madison, WI). The resulting constructs were named pV267 and pV270, and used for DNA immunization. To obtain the quantity of DNA required for all immunizations, *E. coli* DH5α strain (Thermo Fisher Scientific Inc., MA) was transformed by electroporation with the constructs and recombinant plasmid DNA was isolated using the EndoFree Plasmid Giga kit (Qiagen, Valencia, CA).

### Immunization of mice

Mice received, intramuscularly, 100 μg of the pV267 or pV270 constructs (DNA vaccines) in 100 μl of phosphate buffer saline (PBS), divided into two injections of 50 μl in each posterior tibialis muscle. Control groups received either 100 μg pVAX1 in 100 μl of PBS or 100 μl of PBS, injected as described above for the experimental group (Dhama et al., 2008). All groups were immunized three times at 15-day intervals.

### Purification of the protein codified by pV267 and pV270

**Figure 1:**
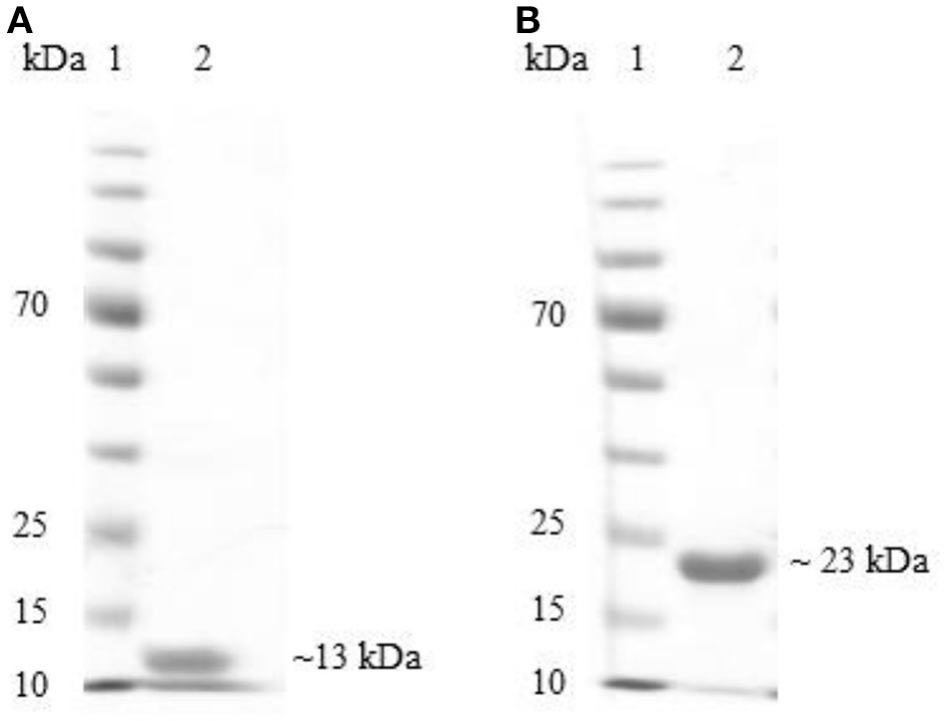
**Electrophoresis of SH3-267 (A) and ZM270 (B) recombinant proteins using 12% sodium dodecyl sulfate polyacrylamide gel (SDS-PAGE)**. Lane 1: molecular weight marker (kDa), lane 2: recombinant proteins.

The proteins codified by the BAB1_0267 and BAB1_0270 ORFs were required to evaluate humoral and cellular adaptive immune responses in experimental and control mice. Bioinformatics analyses indicate that the product of BAB1_0267 includes 117 amino-acids (aa) has a molecular weight (MW) of ~13 kDa, and includes a Src homology 3 (SH3) domain, while the product of BAB1_0270 is a protein of 182 aa with a MW of 22 kDa, corresponding to a Zn-dependent metalloproteinase. These recombinant proteins were expressed in *E. coli* BL21 (DE3) strain and purified by Novoprotein Scientific Inc. (Summit, NJ., USA) and will be hereon named SH3-267 and ZM270, respectively (Figure 1).

### Production of immunoglobulins

The antibody isotypes IgG, IgG1, and IgG2a titers were measured from peripheral blood using an indirect Enzyme-Linked Immunosorbent Assay (ELISA). Serum was collected at days 0, 15, 30 and 45 post-immunization. Ninety-six-well polystyrene microtiter plates (Thermo Fisher Scientific Inc., MA) were coated with 1 μg/ml recombinant SH3-267 or ZM270 proteins in 0.05 M carbonate-bicarbonate buffer (pH 9.6) and left overnight at 4°C in a humid chamber. After washing the plates with PBS-0.05% Tween-20 (v/v) buffer, they were treated with 0.8% gelatin (w/v) in PBS-Tween-20 buffer for 1 h at 37°C in order to block non-specific sites. Serial two-fold dilutions of sera containing primary antibodies from test and control animals were added and incubated for 3 h at room temperature. Rabbit anti-mouse IgG, IgG1, and IgG2a secondary antibodies conjugated with horseradish peroxidase (US Biological, Life Sciences) at a dilution of 1:1000, were added and incubated for 45 min. The reaction was revealed using OPD peroxidase substrate (Sigma-Aldrich Co., MO) and stopped with 50 μl H_2_SO_4_ 2N. Results were read using a VictorX3 ELISA reader (PerkinElmer Chile LTDA, Santiago, Chile) at 490 nm. All assays were done in triplicate.

### Levels of cytokines

To determine the levels of IFN-γ, TNF-α, and IL-4 secreted, it was necessary to prepare cell suspensions from the spleens of experimental and control mice. Briefly, spleens were aseptically removed from mice, disaggregated, re-suspended in Red Blood Cell buffer (Promega, Madison, WI) to eliminate erythrocytes and washed three times using incomplete RPMI 1640 (Thermo Fisher Scientific, MA). Cells were adjusted to a concentration of 4 × 10^6^ viable cells per ml in RPMI 1640 medium supplemented with 10% fetal calf serum (Thermo Fisher Scientific, MA) and antibiotic antimycotic solution (100 UI penicillin, 100 μg/ml streptomycin and 0.25 μg/ml amphotericin B). Cell suspensions were cultured in 24-well plates (Nunc, Denmark) containing 0.8 or 4 μg/ml SH3-267 or ZM270 recombinant proteins and incubated for 72 h at 37°C and 5% CO_2_ to stimulate, *in vitro*, the expression of cytokines. After centrifuging the plates at 400 × g for 10 min, supernatants were collected and cytokines quantified by ELISA sandwich using the Mouse IFN-γ, TNF-α, and IL-4 ELISA kits (eBioscience, San Diego, CA) following the manufacturer's instructions. All assays were performed in triplicate.

### Lymphoproliferation assay

The lymphoproliferative response to the recombinants SH3-267 and ZM270 proteins was evaluated 30 days after the last immunization. Spleens were removed from experimental and control animals and cell suspensions obtained as described above. Cells were adjusted to a concentration of 4 × 10^6^ viable cells/ml and cultured (100 μl per well) in 96-well microtiter plates (Nunc, Denmark), previously sensitized with 0.8 or 4 μg/ml of recombinants proteins or 1.2 or 6 μg/ml *B. abortus* 2308 total proteins (Connolly et al., 2006), and incubated for 72 h at 37°C and 5% CO_2_. After 72 h of incubation, 0.5 μCi/well of thymidine [methyl ^3^H] (20 Ci/mmol; PerkinElmer, MA) was added and 8 h later the samples were placed in vials containing 5 ml of scintillating solution and cells were harvested using filter glass paper (Sigma-Aldrish Co. MO). ^3^H thymidine incorporated was measured using a Beckman LS 6500 Scintillation Counter (Beckman Coulter Inc., CA). Concanavalin A (ConA) (Sigma-Aldrich, MO) at a concentration of 10 μg/ml was used as proliferative positive control and RPMI 1640 supplemented was used as proliferative negative control. Values of negative control were subtracted from the value of all other groups. All assays were done in triplicate.

### Protection experiment

For this assay, mice immunized as described above were challenged, by intraperitoneal injection, with *B. abortus* 2308 at a dose of 10^4^ colony-forming units (CFU) per animal. The RB51 strain commercial vaccine, at a dose of 1 × 10^8^ CFU per mouse, was used as a control (Oñate et al., 2003). After 15 days, all animals were euthanized and their spleens removed. Spleens were homogenized in PBS, serially diluted and cultured in Petri dishes containing agar Columbia supplemented with 5% sheep blood (bioMérieux, Santiago, Chile) for 72 h at 37°C. Bacterial counts were recorded and the number of CFU per spleen calculated. Results are reported as units of protection represented by the difference between mean ± SD of log10 CFU/spleen of the PBS control group with respect to mean ± SD of log10 CFU/spleen values of experimental groups.

### Statistical analysis

The immune response in mice was analyzed using a two-way analysis of variance (ANOVA) and the protective response using a one-way ANOVA. Bonferroni post-test was used to counteract the problem of multiple comparisons. Data were analyzed using Prism 5.0 (GraphPad software). Differences were considered significant if *P* < 0.05.

## Results

### Production of immunoglobulins

**Figure 2:**
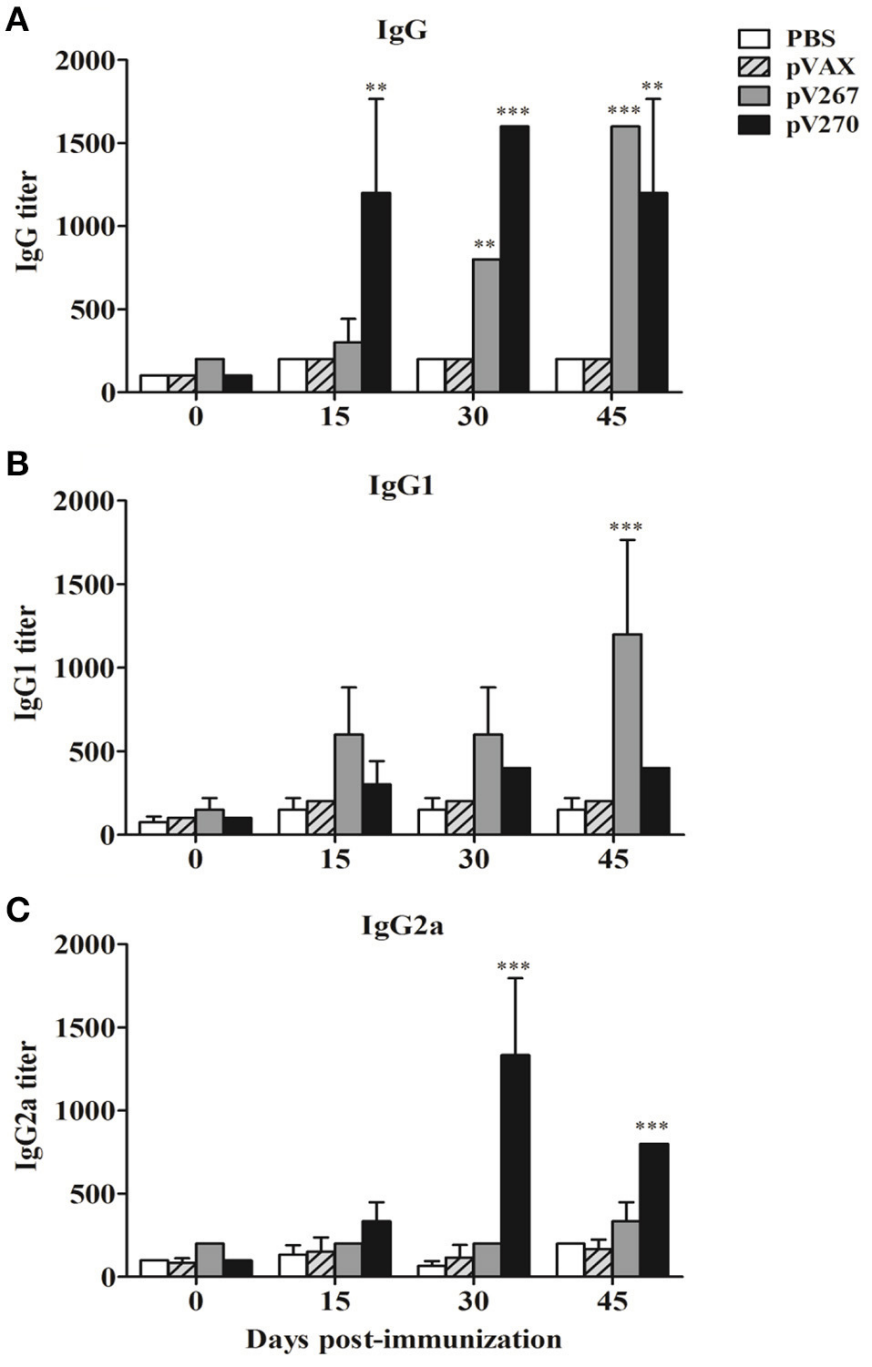
**Serum IgG (A), IgG1 (B), and IgG2a (C) titers measured by ELISA at day 0, 15, 30, and 45 post-immunization (***n*** = 5)**. Results were expressed as mean ± standard deviation (SD) of the last serum dilution yielding a specific optical density higher than the cutoff value. ^**^P < 0.01 and ^***^P < 0.001.

Mice immunized with the pV267 recombinant plasmid had significantly increased titer of IgG at day 30 and 45 post-immunization when were compared to pVAX and PBS control groups (Figure 2A). Furthermore, IgG1 production significantly increased at day 45 post-immunization (*P* < 0.001; Figure 2B), while the IgG2 titer did not alter (Figure 2C). Immunization of mice with the pV270 DNA recombinant plasmid induced raised levels of IgG in all sampled days (*P* < 0.01; Figure 2A). No increment was observed in IgG1 production (Figure 2B), whereas significant increases in titer of IgG2 occurred on day 30 and 45 post-immunization (*P* < 0.001; Figure 2C).

### Cytokine levels

**Figure 3:**
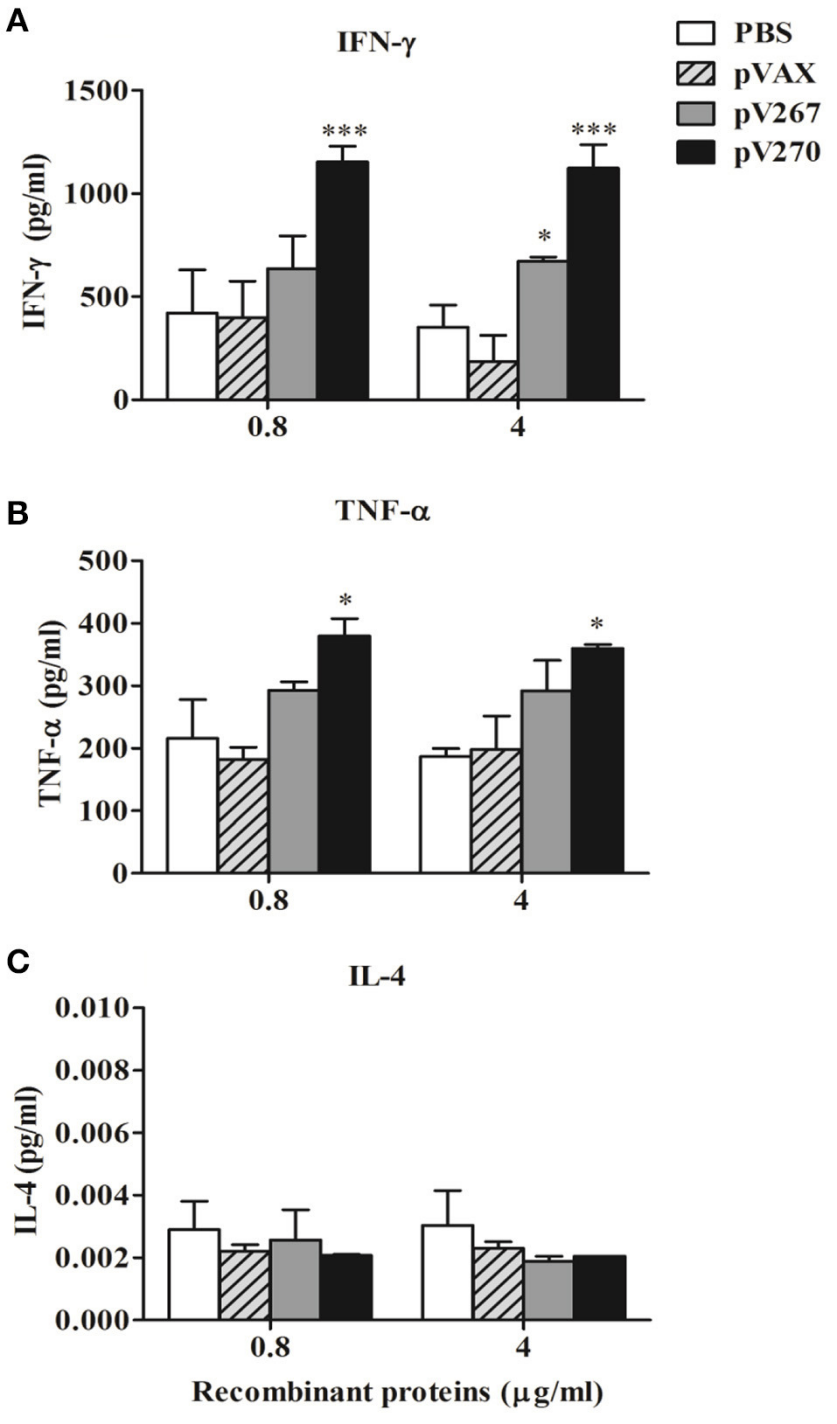
**Expression of IFN-γ (A), TNF-α (B), and IL-4 (C) by spleen cell cultures from mice stimulated with 0.8 and 4 μg/ml of recombinant SH3-267 and ZM270 proteins**. Results were expressed as mean ± SD (*n* = 5). ^*^P < 0.05 and ^***^P < 0.001.

The results demonstrate a significant increase of IFN-γ (*P* < 0.05) in splenocytes obtained from pV267-immunized animals, following their exposure to only 4 μg/ml of SH3-267 recombinant protein (Figure 3A). However, none of the two concentrations (0.8 or 4 μg/ml) increased TNF α levels (Figure 3B). *In vitro* stimulation of splenocytes obtained from pV270-immunized animals incubated with all concentrations of ZM270 induced a significant increase of IFN-γ (*P* < 0.001; Figure 3A) and TNF-α (*P* < 0.05; Figure 3B), not seen in control animals. IL-4 expression (*P* > 0.05) was not affected by any of the SH3-267 or ZM270 recombinant protein concentrations used (0.8 or 4 μg/ml) in animals immunized with the pV267 or pV270 recombinant vectors (Figure 3C).

### Lymphoproliferative response

**Figure 4:**
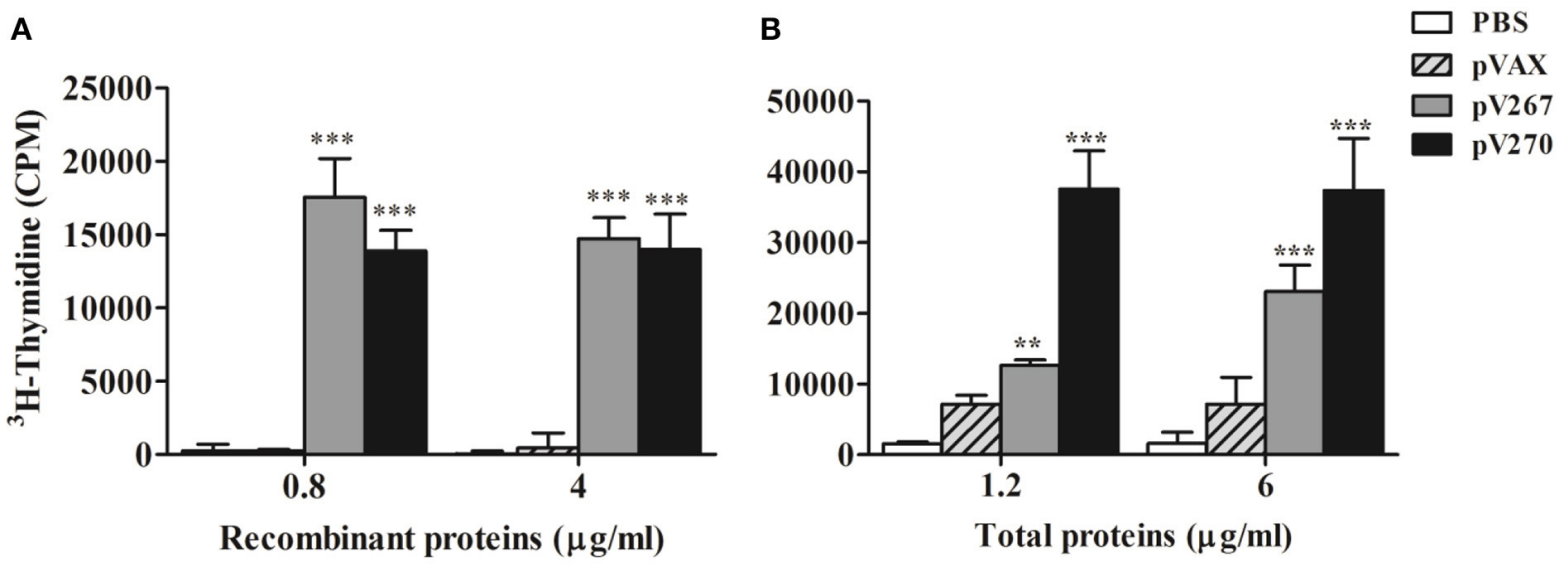
**Lymphocyte proliferation after ***in vitro*** stimulation with (A) 0.8 or 4 μg/ml recombinant proteins and (B) 1.2 or 6 μg/ml ***B. abortus*** 2308 total proteins**. Results are shown as mean ± SD of ^3^H-thymidine incorporation from mice splenocytes (*n* = 5). ^**^P < 0.01 and ^***^P < 0.001.

Proliferation of splenocytes from mice immunized with pV267 DNA vaccine increased significantly after stimulation of splenocytes with 0.8 or 4 μg/ml SH3-267 recombinant protein (*P* < 0.001; Figure 4A). When splenocytes were stimulated *in vitro* with 1.2 or 6 μg/ml *B. abortus* 2308 total proteins, cellular proliferation increased significantly (*P* < 0.001 and *P* < 0.01, respectively; Figure 4B). *In vitro* stimulation of splenocytes from mice immunized with the pV270 DNA vaccine with 0.8 and 4 μg/ml ZM270 recombinant protein product, codified by BAB1_0270 ORF, and 1.2 or 6 μg/ml total proteins of *B. abortus* 2308 also caused increased proliferation of splenocytes (*P* < 0.001; Figures 4A,B). This assay used 10 μg/ml of ConA as positive control, and showed a higher lymphoproliferative response than all experimental groups (data not shown).

### Levels of protection

The protective capacity provided by the pV267 and pV270 DNA vaccines was evaluated by challenging mice immunized with these vaccines with 10^4^ CFU of pathogenic *B. abortus* strain 2308. Results showed that the pV267 DNA vaccine confers protection against the pathogenic agent, as shown by an increase of 0.63 log_10_ units of protection (*P* > 0.05), while immunization with pV270 DNA vaccine afforded 0.91 log_10_ units of protection (*P* < 0.05; Table 1). The vaccinal RB51 strain produced a significant increment of 1.8 log_10_ units of protection compared to control animals (*P* < 0.001).

**Table 1:** **Protection conferred to BALB/c mice immunized with the pV267 and pV270 DNA vaccines when challenged with the pathogenic ***B. abortus*** 2308 strain**.

**Experimental group (*n* = 5)**	**Log_10_ CFU of *Brucella abortus* 2308 per spleen (means ± SD)**	**Log_10_ units of protection^a^**
PBS	5.88 ± 0.06	0
pVAX1	5.88 ± 0.18	0
pV267	5.25 ± 0.15	0.63
pV270	4.97 ± 0.26	0.91^*^
RB51 vaccinal strain	4.01 ± 0.21	1.8^***^

a *units of protection represent the difference between log_10_ CFU values of the PBS control group and the log_10_ CFU values of the immunized group. All animals were challenged with B. abortus 2308 and bacterial counts were assessed at day 15*.

* P < 0.05

and

*** *P < 0.001*.

## Discussion

Since vaccines so far developed to prevent brucellosis are not safe, we evaluated the immunogenicity of recombinant plasmids based on *B. abortus* BAB1_267 and BAB1_0270 ORFs, encoding of a protein with SH3 domain and a Zn-dependent metalloproteinase, respectively, in the search for an effective strategy to prevent this disease. We opted for a DNA immunization strategy because *B. abortus* is a facultative intracellular coccobacilli and recombinant plasmid are able to induce strong cell-mediated immunity (CMI), a response dependent on Th1 CD4+ lymphocytes that express INF-γ, a cytokine pivotal to eliminate this pathogen (Shedlock and Weiner, 2000; Golding et al., 2001).

The observed patterns of serum immunoglobulins and cytokines measured from mice immunized demonstrated that the pV267 recombinant plasmid induced a weak Th1-type immune response, including low levels of IFN-γ and no observed TNF-α (Figures 3A,B), but significant production of IgG1, consist with a Th2-type immune response (Figure 2B; Jankovic and Feng, 2015). Contrastingly, mice immunized with the pV270 recombinant plasmid increasing the titers of IgG2a isotype (Figure 2C), produced by plasma cells in the presence of IFN-γ cytokine (Golding et al., 2001). This response was found to correlate with significant levels of IFN-γ (Figure 3A) and with increased TNF-α production (Figure 3B). Importantly, IFN-γ and TNF-α are associated with effective clearance of intracellular pathogens, demonstrating that immunization with the pV270 recombinant plasmid induced a polarized Th1-type immune response (Golding et al., 2001; Murphy et al., 2001). Humoral immune response induced by the immunization with pV267 or pV270 were correlated with the significant increase of cellular immune response, measured by the lymphoproliferative response of splenocytes after their *in vitro* stimulation with recombinant antigens (SH3-267 or ZM270) and *B. abortus* 2308 total proteins (Figures 4A,B). Furthermore, the lymphoproliferative assay confirmed the *in vivo* expression of the antigen codified by ORFs of *B. abortus* 2308, suggesting that both proteins would be expressed by the pathogen, probably as virulence factors allowing intracellular survival and replication (Ortiz-Román et al., 2014).

The capacity of the immunogenicity induced for recombinant plasmids based on the BAB1_0267 and BAB1_0270 ORFs on protective immunity was evaluated challenging immunized mice with the pathogenic *B. abortus* 2308 strain. Our results confirmed that immunization with pV267 recombinant plasmid induced a weak immunogenicity associated with no significant protection conferred to pV267 immunized mice (Table 1). In eukaryotes, proteins possessing a SH3 domain use this to bind proteins involved in intracellular signaling to facilitate signal transduction, and some can regulate Th2-type immune responses (McCoy et al., 2010). It is possible that the non-significant protection and low production of IFN-γ by this recombinant plasmid is the consequence of the Th2-type response by expression of SH3 domain protein of *B. abortus* in mice. These results demonstrate that IFN-γ production by Th1 subset of CD4 lymphocytes is critical for *B. abortus* clearance (Golding et al., 2001; Murphy et al., 2001). By contrast, immunization with the pV270 DNA recombinant plasmid induced a significant increase of the levels of protection against *B. abortus* 2308 strain, indicating that a polarized pro-inflammatory Th1-type immune response directed against the Zn-dependent metalloproteinase is effective in eliminate *B. abortus* 2308 in a mice model (Table 1). Therefore, considering that Zn-dependent metalloproteinases are used as virulence factors by many pathogens important in public health (Barrett et al., 2004; Bonis et al., 2010; Cafardi et al., 2013; Menon and Govindarajan, 2013), and given their role in intracellular survival and replication of *B. abortus* BAB1_0270 ORF (Bakal and Davies, 2000), we consider Zn-dependent metalloproteinases of *B. abortus* 2308 to be are good target for vaccine design.

## Conclusion

In summary this study shows that using a DNA vaccine codifying a Zn-dependent metalloproteinase is an effective and safe alternative for expressing protecting antigens in BALB/c mice. The vaccine induces a Th1-type polarized immune response and hence protective immunity. The pV270 DNA vaccine is immunogenic and is able to induce a polarized Th1-type response, providing significant levels of protection. It is therefore concluded that this vaccinal candidate is able to induce effective protection against Brucellosis.

## Author contributions

LG, writing and discussion of the result, performs immunization trial, construction of recombinant plasmids, and Protection experiment. FA, conducted antibodies evaluation by ELISA and lymphocyte proliferation assays. PF, evaluation of antibodies and cytokines by ELISA. MF, Culture of bacterial, assay of protection. RM, Lymphocyte proliferation assay. RC, performs evaluation of immune response and statistical analysis of the results. AO, programming and monitoring the experiment, performing the analysis, and discussion and writing the manuscript. AO is principal investigator at the FONDECYT grant that funded this work.

### Conflict of interest statement

The authors declare that the research was conducted in the absence of any commercial or financial relationships that could be construed as a potential conflict of interest. The reviewer SPC and handling Editor declared their shared affiliation, and the handling Editor states that the process nevertheless met the standards of a fair and objective review.
